# Therapeutic Management of Dyslipidemia Patients at Very High Cardiovascular Risk (CARDIO TRACK): Protocol for the Observational Registry Study

**DOI:** 10.2196/resprot.9248

**Published:** 2018-06-29

**Authors:** Poobalan Naidoo, Rashem Mothilal, Dirk Jacobus Blom

**Affiliations:** ^1^ Department of Medical Affairs Sanofi South Africa Johannesburg South Africa; ^2^ Division of Lipidology and Hatter Institute Department of Medicine University of Cape Town Cape Town South Africa

**Keywords:** dyslipidemia, very high cardiovascular risk, maximally tolerated statin, novel lipid lowering therapy

## Abstract

**Background:**

Dyslipidemia is a major modifiable risk factor for atherosclerotic cardiovascular disease. Current South African guidelines recommend titrating lipid-lowering therapy (LLT) to low-density lipoprotein cholesterol (LDL-C) targets stratified by cardiovascular risk. The LDL-C goal for very high-risk patients is <1.8 mmol/L. In international studies, approximately 30% of patients do not achieve this goal despite receiving maximally tolerated statin doses. There is, however, a paucity of data on LDL-C goal achievement in very high-risk South African patients receiving maximal statin doses.

**Objective:**

The goal of the research it to assess LDL-C goal achievement in, and clinical characteristics of, very high cardiovascular risk dyslipidemic patients receiving maximal tolerated statin doses with or without ezetimibe.

**Methods:**

This is an observational, cross-sectional South African registry study that plans to include up to 30 sites and 500 study participants. Adult patients with very high cardiovascular risk status receiving stable, maximally tolerated statin doses (with or without ezetimibe) will be eligible for inclusion.

**Results:**

Funding has been awarded and enrollment began on November 15, 2017, and was completed on April 13, 2018, with 507 participants. Database lock was done on June 21, 2018. The statistical analysis has commenced and we expect the final clinical study report to be completed by October 2018.

**Conclusions:**

This study will document the adequacy of LLT in those at highest risk and will thus fill an important data gap in South Africa. This data may be useful in assessing the need for novel LLTs like proprotein convertase subtilisin/kexin 9 inhibitors that substantially lower cholesterol levels in addition to optimal statin therapy.

**Registered Report Identifier:**

RR1-10.2196/9248

## Introduction

### Background

Atherosclerotic cardiovascular disease is a leading contributor to morbidity and mortality in both developing and developed countries [1-3]. Dyslipidemia is an important modifiable risk factor for atherosclerotic cardiovascular disease and was the risk factor with the highest population attributable risk in the INTERHEART (Effect of Potentially Modifiable Risk Factors Associated with Myocardial Infarction) study [4,5].

The prevalence of dyslipidemia in Africa in general and South Africa specifically is increasing and is probably related to lifestyle changes secondary to rapid urbanization [4,6,7]. Patients classified as very high cardiovascular risk are at greatest risk for either new or recurrent major adverse cardiovascular events. The management of major adverse cardiovascular events consumes significant health care resources in addition to imposing a high societal burden due to frequent loss of productivity and need for care. This is particularly concerning in resource-limited settings where there are multitudes of other health priorities including infectious diseases, interpersonal violence, and trauma. Implementing optimal preventative strategies is thus an important priority for health care in South Africa.

In a registry study conducted in a cardiology subspecialty practice in the United States, 30% of 9950 dyslipidemic patients with coronary artery disease were not at low-density lipoprotein cholesterol (LDL-C) goal despite the prescription of what investigators considered optimal lipid-lowering therapy (LLT) [6]. There is a paucity of South African data exploring lipid goal attainment in very high cardiovascular risk patients receiving optimal LLT, here defined as the prescription of maximally tolerated doses of a statin with or without ezetimibe.

South Africa participated in the Dyslipidemia International Study (DYSIS) [8]. The DYSIS study evaluated lipid target attainment in patients treated with statins and also studied variables affecting lipid control. More than 1000 patients were enrolled in the South African arm, and 50.3% were not at their target LDL-C level. Among very high-risk patients, 73.5% were not at target LDL-C. In this group of patients, only 20.2% were on potency level 4 statins or higher (equivalent to at least simvastatin 40 mg/day). Our study will complement the DYSIS South Africa study by further evaluating the very high-risk patients in whom the primary problem is not prescription of an inadequate statin dose.

The South African arm of the International Cholesterol Management Practice Study (ICLPS) (data on file) study [OBS14286] (an international, cross-sectional, observational study to describe management and LDL-C control versus European Society of Cardiology/European Atherosclerosis Society [ESC/EAS] guidelines of patients receiving lipid-modifying treatments in non-US, non-European countries in real-life) showed that 56% of study subjects were classified as very high cardiovascular risk, and 70% of these patients were not at LDL-C goal (data on file). Almost all (99%) study subjects were treated with a statin, but 75% were not receiving high-intensity statin therapy. The most common reasons participating physicians reported for not escalating patients to higher statin doses were either that they were satisfied with patient’s current dose regimen or that there was a cost issue.

The OBS14286/ICLPS study did not include a sufficient number of patients receiving maximum tolerated statin with or without ezetimibe and was thus unable to provide an accurate estimate of the percentage of very high-risk patients not at goal despite aggressive LLT. Additionally, the number of very high-risk patients not at goal despite optimal LLT was not high enough to allow for reliable patient characterization and identification of factors associated with the inability to reach goal.

### Rationale

This study will describe and quantify the unmet medical need in very high-risk patients on optimal LLT. This group of patients may benefit either from adding ezetimibe to their statin-based therapy or from the prescription of novel LLTs such as proprotein convertase subtilisin/kexin 9 (PCSK9) inhibitors which can lower LDL-C by an additional 50% to 60% on top of statins [9].

Familial hypercholesterolemia (FH) is an autosomal dominant genetic disorder characterized by high concentrations of LDL secondary to defective clearance of LDL by the LDL receptor. FH is highly prevalent in several South African populations secondary to founder effects [10]. Although not all patients with FH are formally classified as very high risk by EAS/ESC guidelines, they were included in this study because many patients with FH are unable to reach target because of their very high baseline LDL-C, and South African guidelines classify FH as a very high-risk condition [6]. FH patients with high LDL-C despite aggressive LLT are also potential candidates for novel therapies.

### Definitions

For the purpose of this study, we defined maximum tolerated statin as either the highest licensed dose of a statin or the highest dose that a patient could tolerate. For patients not at LDL-C goal and not receiving the highest licensed dose of either atorvastatin or rosuvastatin, the reason why the dose was not increased to the highest licensed dose or why a more potent statin was not prescribed needed to be well documented. Acceptable reasons for a patient taking a lower statin dose or a low-potency statin included adverse events on higher doses or concomitant medications that may necessitate lower statin doses (eg, colchicine, amiodarone, digoxin, ranolazine, ticagrelor, sacubitril/valsartan). We considered patients who were not at target and who were not receiving maximal doses of either atorvastatin or rosuvastatin and who had no medically valid reason for not up-titrating as not receiving maximal tolerated statin and thus not eligible for this study.

Maximum intensified LLT was defined as a high-intensity statin, either atorvastatin 40 to 80 mg daily or rosuvastatin 20 to 40 mg daily together with ezetimibe 10 mg daily.

### Study Objectives

#### Primary

The primary objective of this study is to assess the percentage of very high cardiovascular risk and FH patients on maximum tolerated statin with and without ezetimibe not reaching LDL-C goal as defined by the ESC/EAS guideline for the management of dyslipidemia in 2016.

#### Secondary

Secondary objectives of this study are to group patients not at LDL-C goal into those with LDL-C>5 mmol/L, LDL-C 2.5 to 5 mmol/L, and LDL-C 1.8 to 2.49 mmol/L and to explore and compare characteristics of the subjects grouped according to achieved LDL-C, with a particular emphasis on patients with LDL-C>5.0 mmol/L. Characteristics to be explored include age, sex, duration of dyslipidemia, obesity, family history of premature atherosclerotic disease, FH diagnosis, diabetes, hypertension, smoking, and use of combination LLT.

Other secondary objectives are to subgroup patients at LDL-C goal into those with LDL-C<1.8 to 1.0 mmol/L and LDL-C<1.0 mmol/L; determine the percentage of patients not on ezetimibe despite target LDL-C not being reached; determine reasons for nonuse of ezetimibe in patients not at LDL-C goal (eg, cost, physician choice, medical funder refusing coverage); assess percentage of very high cardiovascular risk dyslipidemia patients on maximum intensified LLTs and still not at LDL-C goal and explore characteristics of the subjects with LDL-C>5 mmol/L despite maximum intensified LLTs. Characteristics to be explored include age, sex, duration of dyslipidemia, obesity, family history of premature atherosclerotic disease, FH diagnosis, diabetes, hypertension, and smoking.

## Methods

### Study Design

This is a national, noninterventional, cross-sectional study evaluating LDL-C goal achievement in very high-risk and FH patients receiving maximal tolerated statin therapy. The study will use laboratory data collected during routine care, and no study-specific laboratory investigations will be performed. A single visit coinciding with a scheduled routine medical encounter is planned.

### Selection of Patients

Adult patients with very high cardiovascular risk or FH receiving stable maximum tolerated statin therapy for at least 4 weeks prior to their latest lipid profile are eligible for inclusion. The selection criteria are listed in Textbox 1. We plan to recruit 500 patients in up to 30 centers in South Africa. The minimum patient number is 385 in the stipulated recruitment period of 5 months.

### Selection of Investigators

Potential sites will be evaluated by means of a site feasibility questionnaire and, based on the responses received, sites will be selected to participate. Selection criteria include the potential to recruit the required number of patients within the protocol-specified recruitment period and having adequate time and resources to conduct this study.

### Statistical Considerations

#### Determination of Sample Size

Stata software (StataCorp LLC) was used to determine the sample size for this study. It is estimated that a minimum number of 385 patients will provide over 80% power at the .05 significance level to determine the prevalence of patients in South Africa not reaching LDL-C target levels assuming that 50% to 60% are not at target.

#### Analysis Populations

The analysis population will consist of all patients included in the study who meet all the inclusion criteria and none of the exclusion or withdrawal criteria.

#### Statistical Methods

Qualitative variables will be described by number of observed values, percentage, and number of missing values (patients with missing data will not be included in the percentage calculation). Quantitative variables will be described by number of observed values, mean, standard deviation, median, first quartile, third quartile, minimum, and maximum as appropriate for the distribution.

### Ethical Principles

This study will be conducted in accordance with the principles laid down by the 18th World Medical Assembly and all subsequent amendments and the guidelines for Good Epidemiology Practice. All necessary submissions (eg, institutional review board/independent ethics committee) will be performed in accordance with local regulations including local data protection regulations. This study has received ethics approval by Pharma Ethics and the University of Cape Town Ethics Committee. Site feasibility and the electronic case report form have been completed.

**Selection criteria.**

Inclusion criteria:

- Signed informed consent at enrollment in the study
- Adults aged 18 years and older
- Patient receiving maximum tolerated dose of statin with or without other lipid-lowering therapy (same drugs and stable doses without interruption) for at least 4 weeks prior the latest lipid profile (or at least total cholesterol and low-density lipoprotein cholesterol) up until study entry
- Patient with at least total cholesterol and low-density lipoprotein cholesterol value performed within the past 12 months
- At least one of the following criteria is fulfilled:
  - Previous acute coronary syndrome (ST-segment elevation myocardial infarction, non–ST-segment elevation myocardial infarction, or unstable angina)
  - Coronary revascularization (percutaneous coronary intervention, coronary artery bypass graft surgery, or other arterial revascularization procedures)
  - Stroke or transient ischemic attack
  - Peripheral arterial disease as evidenced by history of either intervention, surgery, amputation, or symptoms with low ankle brachial index <0.9
  - Calculated Systemic Coronary Risk Estimation (Multimedia Appendix 1) ≥10% for 10-year risk of fatal cardiovascular disease
  - Diabetes mellitus with target organ damage such as proteinuria
  - Diabetes mellitus with another major cardiovascular risk factor such as smoking or hypertension
  - Definitive familial hypercholesterolemia as per the Dutch Lipid Clinic Network criteria (Multimedia Appendix 2)

Exclusion criteria:

- Patient currently participating in a clinical trial, compassionate use program, or extended access program
- Patient previously participated in a cholesteryl ester transfer protein or mipomersen clinical trial
- Patient previously participated in a proprotein convertase subtilisin/kexin 9 inhibitor trial and low-density lipoprotein cholesterol taken less than 3 months after the last dose of the proprotein convertase subtilisin/kexin 9 inhibitor
- Clinician suspects poor adherence to lipid-lowering therapy by patient (eg, patient history of poor attendance of scheduled clinic visits, patient admits nonadherence)
- Severe chronic kidney disease (stage IV/V) (ie, estimated glomerular filtration rate <30 mL/min)

Withdrawal criteria:

- Withdrawal of informed consent

## Results

Funding has been awarded and enrollment began on November 15, 2017, and was completed on April 13, 2018, with 507 participants. Database lock was done on June 21, 2018. The statistical analysis has commenced and we expect the final clinical study report to be completed by October 2018.

## Discussion

### Summary

LLT is associated with uniform relative cardiovascular risk reductions across a wide spectrum of patients depending on the LDL-C reduction achieved. However, the absolute risk reduction may vary widely according to baseline absolute risk and LDL-C. The number needed to treat is calculated as 1/absolute risk reduction and is commonly used when evaluating the cost-benefit ratio of novel therapies. Novel and expensive LLT should initially be directed toward patients who have the largest absolute risk reduction and lowest number needed to treat. We thus focused on identifying and characterizing patients with high absolute baseline risk and elevated LDL-C, including FH patients because of their very high LDL-C levels.

The South African lipid management guidelines [6] vary in some respects from the ESC/EAS guidelines [11]. Specifically, the South African guidelines classify all FH patients, even those without evidence of cardiovascular disease, as very high risk, whereas the ESC/EAS guidelines classify the latter group as high risk. Because of the high prevalence of FH in South Africa, all FH patients are classified as very high risk so that they can all access intensive LLTs in a resource-limited setting.

FH will be identified using the Dutch Lipid Clinic Network (DLCN) criteria. To maximize specificity we will only include patients with a DLCN score of greater than 8 or definitive FH. For study participants who do not have a recorded pretreatment LDL-C level, this will be estimated based on the potency of their current LLT (Multimedia Appendix 3).

We excluded patients without overt atherosclerotic cardiovascular disease or one of the other very high-risk indicators but with subclinical atherosclerosis on imaging. This is because these patients probably do not have the same risk as patients who meet the inclusion criteria. Additionally there may be interindividual differences in reporting imaging investigations, especially carotid intima-media thickness assessments.

Patients with severe renal failure are at very high cardiovascular risk, but we excluded them because of the complexity of using LLTs in this group. These patients are frequently prescribed multiple concomitant drugs that may result in drug-drug interactions with statins. Severe renal impairment alters the pharmacokinetics of statins by reducing renal elimination and requires dose reduction to limit deleterious effects including myositis and rhabdomyolysis [12,13]. Furthermore, there is currently no data describing the use of PCSK9 inhibitors in severe renal impairment.

A major challenge was how to exclude subjects who may be nonadherent to pharmacotherapy, as LLT adherence cannot be routinely monitored with drug levels. It is thus best to use other markers that may indicate poor adherence such as poor or incomplete attendance at scheduled clinic visits, failure to refill prescriptions, or patient reported nonadherence. Unfortunately, objectively determining adherence remains challenging and some patients entered into the study may still be non- or incompletely adherent despite the exclusion criteria.

Patients who had previously participated in a cholesteryl ester transfer protein or mipomersen clinical trial were excluded because of the long elimination half-lives of these 2 drugs and the risk of residual effects on the lipid profile.

We excluded patients who had taken a PCSK9 inhibitor within the last 3 months to eliminate the possibility of carry-over effects. PCSK9 inhibitors were not commercially available in South Africa when this study was conceived, and all exposure to such drugs would thus have been either via a clinical trial or compassionate use. It was important not to exclude patients with prior PCSK9 inhibitor exposure as many of these patients would likely be candidates for these drugs once they are commercially available in South Africa.

### Limitations

This observational registry study has limitations. Trial sites were conveniently sampled and there may thus be selection bias. This study will largely be done in urban centers at private health care facilities and will thus not be fully representative of the entire South African population.

### Conclusion

This study will fill an important data gap by characterizing the highest atherosclerotic cardiovascular disease risk populations in South Africa and providing data on the unmet need for additional LLT.

## Appendix

Multimedia Appendix 1 Systemic Coronary Risk Estimation.

Multimedia Appendix 2 Dutch Lipid Clinic Network criteria.

Multimedia Appendix 3 Drug conversion factors.

## Abbreviations

DLCN: Dutch Lipid Clinic Network
EAS: European Atherosclerosis Society
ESC: European Society of Cardiology
FH: familial hypercholesterolemia
ICLPS: International Cholesterol Management Practice Study
LDL-C: low-density lipoprotein cholesterol
LLT: lipid-lowering therapy
PCSK9: proprotein convertase subtilisin/kexin 9
