# Supplementary figures and images for: Therapeutic Management of Dyslipidemia Patients at Very High Cardiovascular Risk (CARDIO TRACK): Protocol for the Observational Registry Study
Source: JMIR Res Protoc. 2018 Jun 29;7(6):e163. doi: 10.2196/resprot.9248 (PMC6045791; doi:10.2196/resprot.9248)

# Appendix 1

## SCORE Chart

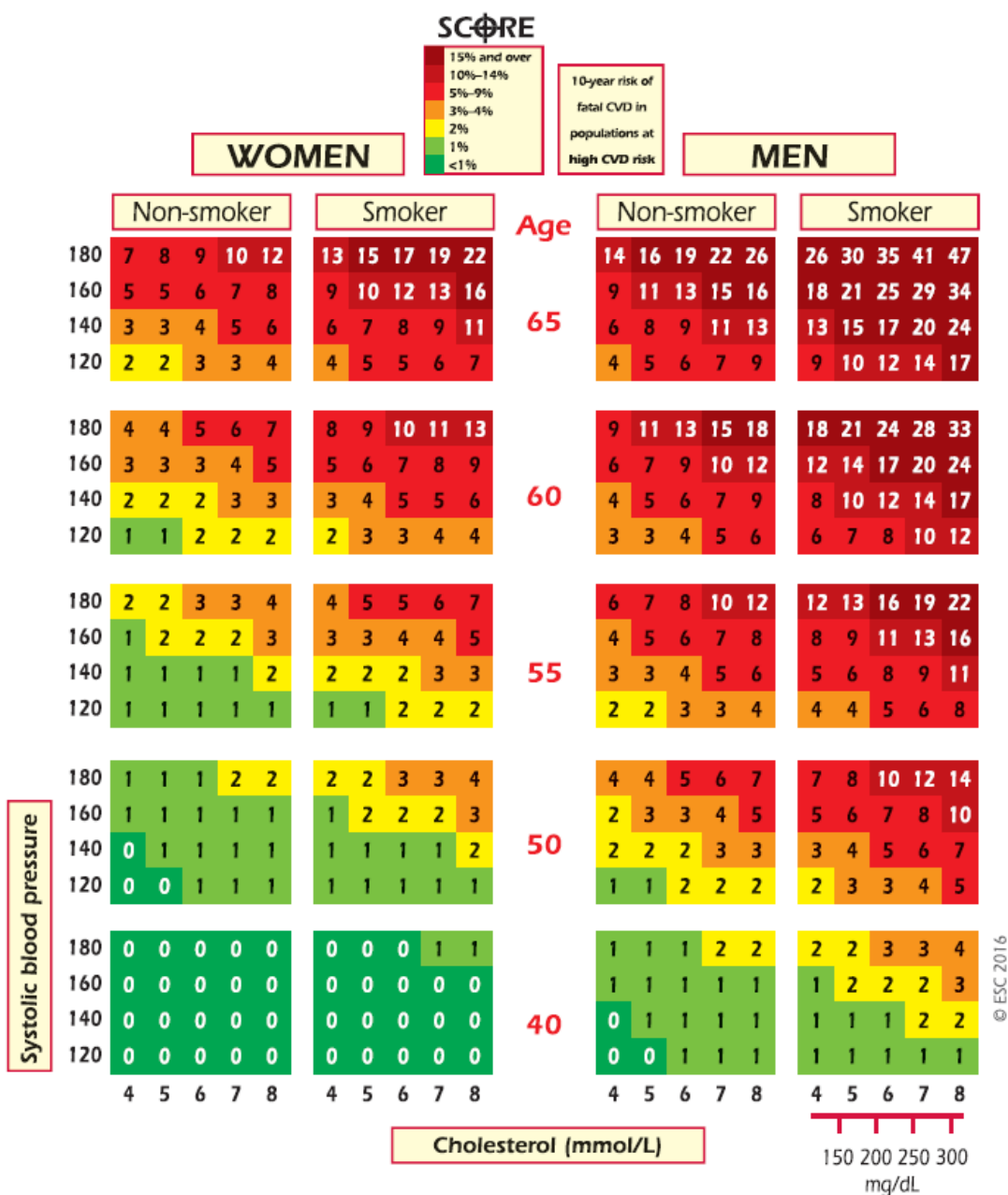

Supplement: Multimedia Appendix 1 [file resprot_v7i6e163_app1.pdf]
