# Supplementary material for: Therapeutic Management of Dyslipidemia Patients at Very High Cardiovascular Risk (CARDIO TRACK): Protocol for the Observational Registry Study
Source: JMIR Res Protoc. 2018 Jun 29;7(6):e163. doi: 10.2196/resprot.9248 (PMC6045791; doi:10.2196/resprot.9248)
Supplement: Multimedia Appendix 2 [file resprot_v7i6e163_app2.pdf]

Appendix 2

The Dutch Lipid Clinic Network Diagnostic Criteria

Criteria	Score
First-degree relative with premature coronary and/or vascular disease (men ≤55 years, women ≤60 years), OR	1
First-degree relative with known LDL-cholesterol ≥95th percentile for age and sex	
First-degree relative with tendon xanthomata and/or arcus cornealis, OR	2
Children aged ≤18 years with known LDL-cholesterol ≥95th percentile for age and sex	
Patient with premature coronary artery disease (age as above)	2
Patient with premature cerebral or peripheral vascular disease (age as above)	1
Tendon Xanthomas	6
Arcus cornealis at age ≤45 years	4
LDL Cholesterol (mmol/L) (mg/dL)	
LDL-C ≥8.5 (330)	8
LDL-C 6.5 - 8.4 (250 - 329)	5
LDL-C 5.0 - 6.4 (190 - 249)	3
LDL-C 4.0 - 4.9 (155 - 189)	1
DNA Analysis – functional mutation LDLR, APOB and PCSK9	8
Stratification	Total Score
Definite Familial Hypercholesterolemia	>8
Probable Familial Hypercholesterolemia	6-8
Possible Familial Hypercholesterolemia	3-5
Unlikely Familial Hypercholesterolemia	<3
