# Supplementary material for: Therapeutic Management of Dyslipidemia Patients at Very High Cardiovascular Risk (CARDIO TRACK): Protocol for the Observational Registry Study
Source: JMIR Res Protoc. 2018 Jun 29;7(6):e163. doi: 10.2196/resprot.9248 (PMC6045791; doi:10.2196/resprot.9248)
Supplement: Multimedia Appendix 3 [file resprot_v7i6e163_app3.pdf]

Appendix 3
Conversion factors

Drug/Drugs	Factor
Pravastatin	
10 mg	1.2
20 mg	1.3
40	1.5
80	1.5
Pravastatin / Ezetimibe	
10 / 10 mg	1.5
20 / 10 mg	1.6
40 / 10 mg	1.7
Simvastatin	
10	1.4
20	1.6
40	1.7
80	1.9
Simvastatin /Ezetimibe	
10 / 10	1.9
20 / 10	2.0
40 / 10	2.3
80 / 10	2.4
Atorvastatin	
10	1.6
20	1.8
40	2.0
80	2.2
Atorvastatin / Ezetimibe	
10 / 10	2.0
20 / 10	2.2
40 / 10	2.2
80 / 10	2.5
Rosuvastatin	
5	1.8
10	1.9
20	2.1

40	2.4
80	2.4
Rosuvastatin / Ezetimibe	
10 / 10	2.5
20 / 10	2.7
40 / 10	3.3
Fluvastatin	
10	1.2
20	1.3
40	1.4
80	1.5
